# Assessment of Patients’ Confidence Regarding a New Triage Concept in a Medical Retina Clinic during the First COVID-19 Outbreak

**DOI:** 10.3390/ijerph18115846

**Published:** 2021-05-29

**Authors:** Anahita Bajka, Maximilian Robert Justus Wiest, Timothy Hamann, Mario Damiano Toro, Sandrine Anne Zweifel

**Affiliations:** 1 Department of Ophthalmology, University Hospital of Zurich, 8091 Zurich, Switzerland; anahita.bajka@usz.ch (A.B.); maximilian.wiest@usz.ch (M.R.J.W.); timothy.hamann@usz.ch (T.H.); mario.toro@usz.ch (M.D.T.); 2 University of Zurich, 8006 Zurich, Switzerland; 3 Chair and Department of General and Pediatric Ophthalmology, Medical University of Lublin, 20079 Lublin, Poland

**Keywords:** age-related macular degeneration, anti-VEGF, COVID-19 outbreak, diabetic macular edema, emergency regime, eye care, intravitreal injection, patients’ confidence, retinal vein occlusion, triage

## Abstract

Background: During the first COVID-19 pandemic outbreak, a new triage concept had to be implemented for patients with retinal diseases having a scheduled appointment at the medical retina clinic. In this study, we aimed to assess patients’ confidence in this triage concept and patients’ satisfaction regarding the received treatment during the outbreak. Methods: This retrospective study included all patients with a diagnosed retinal disease, triaged into three priority groups based on their condition’s urgency during lockdown. After restrictions were eased, a subset of previously triaged patients was interviewed to assess their confidence in the triage and their satisfaction regarding the received treatment during the pandemic. Results: In total, 743 patients were triaged during the lockdown. Over 80% received an urgent appointment (priority 1). Among all priority 1 patients, over 84% attended their appointment and 77% received an intravitreal injection (IVI), while 7% cancelled their appointment due to COVID-19. In post-lockdown interviews of 254 patients, 90% trusted the emergency regimen and received treatment. Conclusions: Our triage seemed to be useful in optimizing access to treatment for patients with retinal diseases. An excellent rating of patients’ confidence in the triage and satisfaction regarding the received treatment during the first COVID-19 outbreak could be achieved.

## 1. Introduction

The coronavirus disease 2019 (COVID-19) pandemic has had a major impact on healthcare systems around the world, necessitating substantial changes in social and hygienic measures, which have led to significant issues in access to eye care [1,2,3,4]. Since certain ophthalmological examinations require close contact with patients (e.g., slit-lamp examinations), the possibility of SARS-CoV-2 transmission during these examinations cannot be ignored [5,6,7]. Thus, many ophthalmological societies have recommended avoiding all treatments except urgent or emergent care and to limit the exposure time in the hospital in order to reduce the risk of SARS-CoV-2 transmission [4,8,9].

Intravitreal injection (IVI) treatment in ophthalmological centers has become one of the most common treatments for several retinal diseases, such as neovascular age-related macular degeneration (nAMD), central and branch retinal vein occlusions (CRVO and BRVO), and proliferative and non-proliferative diabetic retinopathy (PDR and NPDR) with diabetic macular edema (DME), secondary choroidal neovascularization (CNV), leading to significant improvements in treatment possibilities for these patients [10,11,12]. The appropriate timing and number of IVIs has been shown to determine long-term visual outcomes [8,13,14]. For instance, the early treatment of nAMD patients leads to better visual acuity outcomes [5,6]. Conversely, delayed or suspended essential ophthalmological treatment may cause rapid and substantial vision impairment or even irreversible blindness [5,15,16]. An increase in the rates of vision impairment among patients receiving IVI treatment could lead to long-term undesirable effects on quality of life, including daily activities, social interactions, and work opportunities. Especially in the elderly population, the risk of accidents and injuries increases with progressive vision loss [17,18,19], increasing the long-term social costs due to more elders being in need of care [20,21].

To avoid these circumstances, the risk–benefit profiles of patients with retinal diseases have to be determined, particularly in the elderly population, who are more vulnerable to COVID-19. A triage to identify patients with diseases in potentially irreversible sight-threatening stages may improve retinal disease care during the pandemic [8,22], and is crucial since patients may be asymptomatic in the early stages of a retinal disease, when vision loss is often treatable or even avoidable through appropriate measures. Therefore, ophthalmologists practicing IVI treatment should be supported to make guided decisions [1,8,17,22,23]. However, despite the extensive literature describing the impact of COVID-19 on ophthalmological treatment protocols, there seem to be no studies on a triage system for an IVI emergency regimen during the COVID-19 outbreak.

In this study, we investigated patients’ confidence in a triage concept and patients’ satisfaction regarding the received treatment in our medical retina clinic during the first COVID-19 outbreak in Switzerland. Furthermore, we aimed to investigate if their objective visual acuity and subjective visual function had an influence on their confidence and satisfaction. The triage was based on a review of electronic health records (eHRs) and was used to prioritize access to IVI treatment for patients with a retinal disease during the first COVID-19 outbreak in Switzerland between 16 March 2020 and 26 April 2020.

## 2. Materials and Methods

This was a retrospective study of regular patients with a diagnosed retinal disease at the Department of Ophthalmology, University Hospital Zurich, Switzerland. Our research project did not fall within the scope of the Human Research Act, so the approval of the Ethics Committee was not required.

The Swiss government had announced an “extraordinary situation” on 16 March 2020, which led to a lockdown, where people were still allowed to leave their homes as they pleased. However, almost every public institution, all restaurants, bars and all stores except grocery shops were closed. According to the new regulations, outpatient appointments had to be carefully reassessed, prioritizing access to urgent and emergent cases. Thus, an emergency regimen had to be established for the injection clinic: a triage was set up by medical retina specialists to assess patients with a scheduled appointment between 16 March 2020 and 26 April 2020 [1,8,17,22,23].

Therefore, the eHRs of each patient were reviewed by two well-trained and certified retina specialists to assess the patients’ general and ophthalmological condition. In cases of disagreement, a third investigator was consulted for the final decision. The triage categorized patients into three groups (priority 1 to priority 3) based on the urgency of their treatment. For each group, the retinal specialists assessed the suspected vision loss if the planned treatment was postponed. When an irreversible vision loss was expected within 1 to 2 months if treatment was withheld, patients were assigned to the first group (priority 1). This group included mainly patients with an active nAMD under a treat-and-extend regimen and a planned appointment during lockdown, acute retinal vein occlusions and other newly diagnosed retinal disorders where an anti-VEGF therapy was indicated. Progression of the disease in the last visits was also a criteria to treat patients as planned. In the second group (priority 2), all patients were included if no irreversible vision loss was expected within 3 months in the case of postponed treatment. This group included nAMD patients under a treat-and-extend regimen with long intervals and without signs of activity in the last visit. When no vision loss within 3 months or more was suspected in case of a withheld treatment, patients were assigned to the third group (priority 3). This group contained for example AMD patients without signs of an active neovascularization and not having received any treatment within the last visits.

In general, each case had to be assessed individually, weighing up the risk of vision loss against the risk of patients’ exposure during the lockdown. Subsequently, patients were scheduled for urgent (priority 1) or extendable (priority 2) visits or put on a waiting list (priority 3). To limit exposure time in the hospital, all priority 1 patients received an initial check to exclude a red eye or a recent vision loss since their last visit. Patients showing one of these findings underwent a clinical examination to investigate acute conditions and to reconsider the indications for IVI treatment, and those without these findings received IVI treatment directly without visual acuity and imaging (optical coherence tomography (OCT)) evaluations, which are usually performed at every visit in our clinic. Priority 2 and 3 patients received a phone call and/or a letter from the clinic that included information about their postponed appointment and advised them to call our clinic immediately in the case of a decrease in visual acuity or emergency symptoms.

Safety and hygiene measures were employed to reduce the risk of infection, including (1) fewer scheduled appointments per time unit to reduce the number of patients coming to the facility at the same time; (2) ensuring that accompanying people waited outside of the hospital; and mandating (3) distance and (4) mask-wearing for both healthcare workers and patients.

After the lockdown, from May to August 2020, all patients were asked during their regular visit in the clinic if they want to participate in an interview, which aimed to determine patients’ confidence in the triage and patients’ satisfaction regarding the received treatment at our department during the lockdown. The interview questions were based on the “Visual Function Questionnaire—25” (VFQ-25, Questionnaire S1) and an additional questionnaire including 11 questions focusing on patients’ confidence regarding the received treatment (COVIDQ, Questionnaire S2) during the pandemic. Each interview was held by the same interviewer in German, or English if required, and took approximately 10 min for both questionnaire assessments.

The VFQ-25 is a standardized questionnaire containing 25 questions regarding self-reported vision-targeted health status, and is of great importance for patients with chronic eye diseases. The questions assess patients’ estimation regarding the impact of their visual impairment on their daily life. Most of the questions have a grading system from “very poor” up to “excellent” containing 5 to 6 answer options. The 11 further questions were developed by the medical retina team at the USZ to assess patients’ confidence in the triage and patients’ satisfaction regarding the received treatment at the medical retina clinic during the COVID-19 pandemic. The questions assessed topics such as whether patients were afraid of vision impairment due to the changes in treatment during the lockdown and how they perceived their visit during the lockdown.

All patients with severe hearing difficulties or any cognitive impairment that could interfere with an interview were excluded. All patients provided informed consent to be interviewed. Before and after the lockdown, all patients underwent a complete ophthalmological evaluation, including measurements of best-corrected visual acuity (BCVA), intraocular pressure (IOP), examination of the anterior segment and dilated fundus, as well as OCT imaging. BCVA was measured using Early Treatment Diabetic Retinopathy Study (ETDRS) charts by a single well-trained and experienced ophthalmologist. Spectral domain OCT images were acquired with a Heidelberg Spectralis device (version 1.9.10.0) running the Heidelberg software (Spectralis Viewing Module 6.0.9.0; Heidelberg Engineering). In diabetic patients, center subfield thickness (CST) was evaluated for each eye, and a value of at least 290 μm in women and at least 305 μm in men was defined as DME [24]. In addition, DME was classified as center-involving (CI-DME) if intraretinal fluid was within a 1 mm diameter circle centered at the fovea [25].

Data regarding age, sex, main ophthalmic diagnosis, and BCVA for both eyes were collected from electronic medical records.

### Statistical Analysis

Data regarding age, sex, main ophthalmic diagnosis and BCVA, and all answers of the VFQ-25 as well as the 11 additional questions, were descriptively analyzed. All variables are reported as mean values ± standard deviation (SD). Two-sided *p*-values less than or equal to 0.05 were considered statistically significant. Analysis of variance (ANOVA) and chi-squared tests were performed. The data were analyzed using the Statistical Package for the Social Sciences for Windows (v.17.0; SPSS, Chicago, IL, USA).

## 3. Results

In total, 743 patients were triaged during the aforementioned period. The triaged patients consisted of 369 women and 374 men; mean age was 74 years (SD, ±14; range, 17–98 years). The triage grouped the patients as follows: priority 1, 597 patients; priority 2, 110 patients; priority 3, 36 patients. The frequency of primary ophthalmic diseases by priority group is listed in Table 1.

The study protocol was reviewed and approved by the Kantonale Ethikkomission (KEK) of Zurich. As this was a retrospective review of records, the KEK gave this study exempt status and waived the requirement of informed consent.

**Figure 1:**
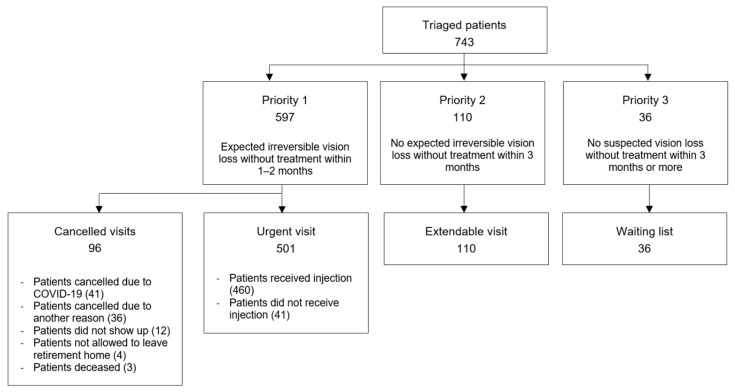
Flowchart outlining the triage used at the medical retina clinic, Department of Ophthalmology, University Hospital of Zurich. Each square contains the number of patients and further descriptions for each group.

Of all patients triaged as priority 1, 96 did not attend the planned urgent visit, of whom 41 cancelled their appointment due to the COVID-19 pandemic (Figure 1). Among the 501 patients who attended their urgent visit, 460 received IVI treatment, of whom 25 received two injections within the lockdown.

**Figure 2:**
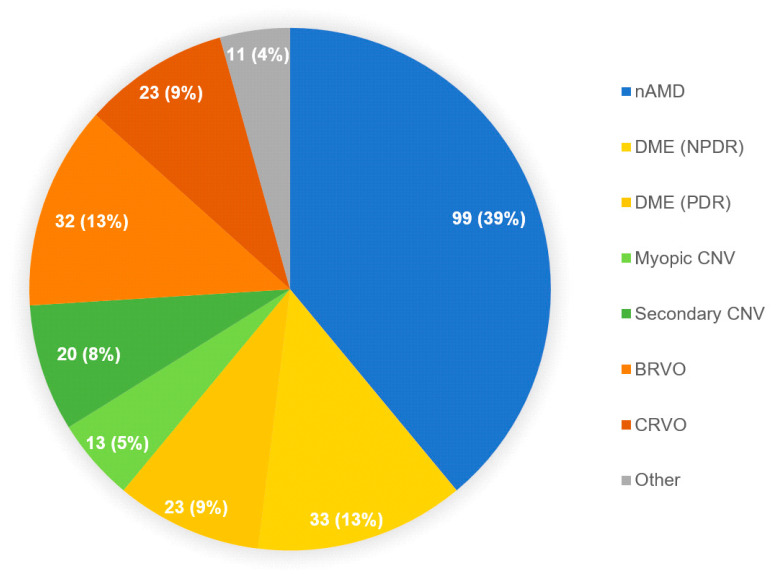
Primary ophthalmic diseases of the interviewed study participants. The graph shows the number of patients in each group followed by its percentage among all primary ophthalmic diseases. Abbreviations: nAMD: neovascular age-related macular degeneration; BRVO: branch retinal vein occlusion; CRVO: central retinal vein occlusion; Myopic CNV: myopic choroidal neovascularization; Secondary CNV: secondary choroidal neovascularization, other than AMD or myopia-related; DME: diabetic macular edema; NPDR: non-proliferative diabetic retinopathy; PDR: proliferative diabetic retinopathy.

Among all triaged patients, 254 (112 women and 142 men) were interviewed from May 2020 to August 2020. The distribution of primary ophthalmic diseases is shown in Figure 2.

**Figure 3:**
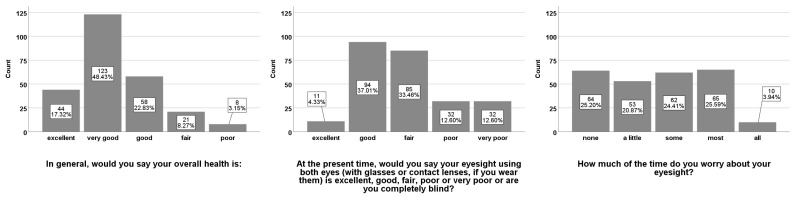
Results from the Visual Field Questionnaire—25 (VFQ-25) assessments. Answers to the questions, “In general, would you say your overall health is:”, “At the present time, would you say your eyesight using both eyes (with glasses or contact lenses, if you wear them) is excellent, good, fair, poor or very poor or are you completely blind?” and “How much of the time do you worry about your eyesight?” Results for each bar are shown in absolute numbers and percentages.

Mean BCVA (±SD) of the right eye (BCVA.OD) after lockdown was 64.6 (±24) ETDRS letters and 65.3 (±25) ETDRS letters for the left eye (BCVA.OS). Mean CST (±SD) for all female patients with DME was 293.2 (±56) μm at the right eye and 291.4 (±57) μm at the left eye. Mean CST (±SD) for all male patients with DME was 312.5 (±102) μm at the right eye and 304.5 (±75) μm at the left eye. Out of all DME patients, 50% had a CI-DME on the right eye and 52% on the left eye. Among all questioned patients, 156 (61.4%) received IVI treatment during the emergency regimen. In the VFQ-25 assessments, 167 patients (66%) described their overall health as “excellent” or “very good”, 115 (45%) patients described their binocular eyesight as “excellent” or “good”. To the question “How much of the time do you worry about your eyesight?”, 64 (25%) patients answered with “none”, 53 (21%) answered “a little”, 62 (24%) responded with “some”, 65 with “most” (26%), and 10 with “all” (4%) of the time (Figure 3).

**Figure 4:**
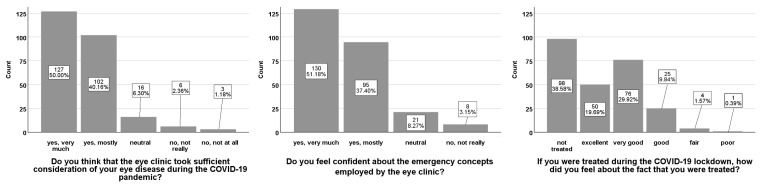
Results from the additional questionnaire assessments. Answers to the questions “Do you think that the eye clinic took sufficient consideration of your eye disease during the COVID-19 pandemic?”, “Do you feel confident about the emergency concepts employed by the eye clinic?”, “If you were treated during the COVID-19 lockdown, how did you feel about the fact that you were treated?” Results for each bar are shown in absolute numbers and percentages.

When asked if they were worried about their eyesight due to the general postponement of non-urgent/emergency procedures during the lockdown, 90 (35%) patients answered with “rather not” and 132 (52%) patients answered “not in the least”. On the question of how well they felt informed about the prognosis of their ophthalmological treatment by the Department of Ophthalmology, 79 (31%) patients answered with “very good” and 123 (48%) patients answered “good”, while 52 (20%) patients answered “adequately” (26; 10%) or worse (“sufficient”, 12 (5%); “bad”, 14 (6%)). In total, 229 (90%) patients answered “very much” (127; 50%) or “yes, mostly” (102; 40%) in response to the question assessing whether the Department of Ophthalmology paid due diligence to their ophthalmic disease. Regarding the emergency regimen of the medical retina clinic, 225 (89%) patients used “very much” or “mostly” to describe their confidence in the regimen. Of all 156 interviewed patients who received IVI treatment during the lockdown, 126 (81%) felt “excellent” (50; 32%) or “very good” (76; 49%) about being treated (Figure 4).

**Figure 5:**
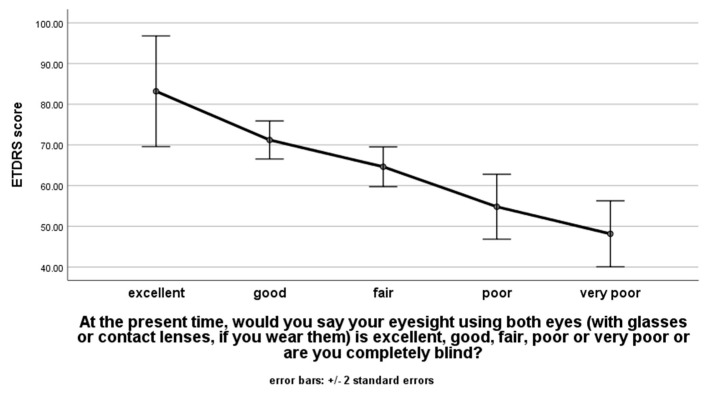
ANOVA analysis of the best corrected visual acuity of the right eye (BCVA.OD) with ETDRS letter scores in comparison with their self-estimated visual acuity from the answer to the question “At the present time, would you say your eyesight using both eyes (with glasses or contact lenses, if you wear them) is excellent, good, fair, poor or very poor or are you completely blind?” Error bars with ± 2 standard errors.

ANOVA showed that patients with better ETDRS letter scores had significantly higher self-classified eyesight (*p* < 0.001) (Figure 5). No statistically significant difference in ETDRS letter scores and subjective visual function was observed between different grades of patients’ satisfaction regarding the received treatment (*p* = 0.933 and *p* = 0.572, respectively). The chi-squared test comparing patients’ trust in the Ophthalmological Department’s emergency planning showed no relationship between patients being either classified as treated or deferred during triage and their trust in the emergency planning (*p* = 0.334).

## 4. Discussion

In this study, we assessed patients’ confidence in a triage modality and patients’ satisfaction with their received treatment at our medical retina clinic during the first lockdown in Switzerland from 16 March to 26 April. Out of 734 triaged patients, over 80% received an urgent appointment (priority 1). Among all priority 1 patients, over 84% attended their appointment and 77% received an IVI, whereas 7% cancelled their appointment due to COVID-19. Our results have shown that out of 254 post-lockdown interviewed patients, 90% were confident in the emergency regimen and were satisfied with the received treatment.

In our cohort, the majority of priority 1 and priority 2 patients had nAMD, including patients with stable diseases in a treat-and-extend regimen, followed by CRVO. However, most priority 3 patients had DME (Table 1). Some guidelines recommend the prioritization of patients with nAMD, neovascular glaucoma, new CRVO, any sudden significant vision loss, and monocular status [2,17]. Indeed, patients with BRVO or DME (with NPDR or PDR) show irreversible vision loss in the short term less frequently [26]. Thus, postponed appointments can be considered for non-monocular patients with DME or BRVO, unless they had significant recent vision loss or are in an acute phase of their disease. This approach could reduce patients’ exposure in clinics, which is especially pertinent for diabetic and elderly patients [27,28]. Thus, the outcomes of the triage at our medical retina clinic were consistent with the aforementioned guidance to prioritize nAMD and acute retinal vein occlusions over DME for urgent visits.

According to our results, most patients (90%) were confident in the triage system and were satisfied with the received treatment during the lockdown. It was shown in patient’s satisfactory surveys that communication, empathy and caring from the hospital are important determinants of patients’ satisfaction [29,30]. High patient satisfaction is important in improving healthcare services and for acceptance and adherence to a new triage system, as described above [31].

Many ophthalmological specialists have provided recommendations and guidelines for IVI treatment during the COVID-19 pandemic [1,8,17,22,23]. To date, there is a consensus that IVI treatment during the COVID-19 pandemic has decreased [32]. However, delays in delivering the intended treatment can severely affect patients’ clinical outcome [5,8]. At our department, the number of IVIs during the COVID-19 lockdown showed only a slight reduction of 8.3% (502/460) when compared to the same period in 2019.

Patients in the early stages of a retinal disease may be asymptomatic, and vision loss at this stage is often treatable or even avoidable through appropriate measures. Moreover, a two-month delay in the initial injection can results in a loss of one line of ETDRS letters [8]. Therefore, as our triage also suggests, new patients with possible nAMD or other newly diagnosed retinal diseases that need new IVI treatment should not be neglected.

As the mean age of our treated patients was over 70 years, the patients were more likely to show severe progression of a SARS-CoV-2 infection. However, studies comparing the risk of SARS-CoV-2 infections among nAMD patients with possible visual impairment showed that reduced disease burden through IVI treatment outweighed the expected loss of health-adjusted life-years from a possible SARS-CoV-2 infection [33,34]. Using our triage modality, no patient or healthcare worker was infected by SARS-CoV-2 at our medical retina clinic. Therefore, it seems that our triage system has provided a safe option to reschedule appointments during the emergency regimen, thus limiting access when not necessary, especially in aged individuals with comorbidities. Thus, whenever possible, IVI treatment should be continued under strict execution of the aforementioned measures.

The ongoing COVID-19 pandemic has led to the creation of numerous new triage systems, which have raised ethical debates. Any triaging that assesses the urgency of patients’ treatment should be based on the principle of bioethics while weighting equity and equality [16,19,35,36]. While equality is fundamental to everyday practice of medicine, under certain circumstances, patients in greater need have to be prioritized over others depending on the urgency and potential adverse outcomes, which can nevertheless lead to imbalances in patient treatments. These considerations should be reviewed considering the local environment, the epidemiological situation, and the capacity for rescheduling postponed appointments. The risks should be discussed with the patients to ensure they are informed and comfortable with the treatment approach.

Our triage system was primarily based on eHRs’ review, and as our review process required up to three clinicians, the triage might be limited and less reliable for smaller or solo-practices. To not increase patients’ exposure additionally and to have the most comparable set of patients’ feedback, we decided to do only face-to-face interviews at patients’ regular visits. Nevertheless, there was no selection made on site, and all patients were asked to participate in an interview. As an additional limitation, some subgroups only contained a small number of patients, limiting the generalizability of our subgroup analysis, and due to its explorative and retrospective design, no sample size calculation was performed. Therefore, it cannot be ruled out that with a larger dataset, significant associations can be found. Furthermore, we analyzed patients’ confidence shortly after the implementation of the emergency regimen; therefore, the long-term influence of a lockdown on clinical outcomes and patients’ confidence and satisfaction remains uncertain.

Despite all the limitations, our study may help policymakers in prioritizing patients for IVI treatment during future pandemic outbreaks. Indeed, while we are treating patients every day with our best evidence-based knowledge, we should not forget to ask our patients, being most affected by our decisions, how they feel about the management in our clinic during an exceptional situation such as a lockdown, which will probably occur more often in the future of this ongoing COVID-19 pandemic. Further randomized trials on a larger sample size and with a longer follow-up are needed to show the impact of this triage system on clinical outcomes and patients’ confidence and satisfaction in the long-term.

## 5. Conclusions

According to our results, patients trusted the implemented triage and were satisfied with the received treatment in our medical retina clinic. Within the first COVID-19 pandemic outbreak, our triage modality seemed to have helped in prioritizing urgent cases, such as patients with an acute retinal vein occlusion, patients with a new diagnosis that needed IVI treatment, and patients with an active nAMD in a treat-and-extend-regimen.

To date, data to predict the course of the COVID-19 pandemic in the near future are limited, and many countries have reinitiated lockdown measures to avoid a next pandemic wave. Efforts to optimize access to eye care while broadly maintaining SARS-CoV-2 infection control may lead to patients’ stable visual acuity, which is mandatory for patients with retinal diseases to maintain independent everyday living.

## Figures and Tables

**Table 1:** Primary ophthalmic diseases of patients in numbers and percentage for each priority group.

Primary Ophthalmic Diseases	Priority 1 (*n*) (%)	Priority 2 (*n*) (%)	Priority 3 (*n*) (%)
nAMD	296 (49.6)	57 (51.8)	8 (22.2)
BRVO, CRVO	115 (19.3)	18 (16.4)	6 (16.7)
DME (NPDR and PDR)	98 (16.4)	24 (21.8)	17 (47.2)
Secondary CNV	35 (5.9)	5 (4.6)	1 (2.8)
Myopic CNV	22 (3.6)	3 (2.7)	1 (2.8)
Other	31 (5.2)	3 (2.7)	3 (8.3)
Total	597 (100)	110 (100)	36 (100)

Abbreviations: nAMD: neovascular age-related macular degeneration; BRVO: branch retinal vein occlusion; CRVO: central retinal vein occlusion; myopic CNV: myopic choroidal neovascularization; secondary CNV: secondary choroidal neovascularization, other than AMD or myopia-related; DME: diabetic macular edema; NPDR: non-proliferative diabetic retinopathy; PDR: proliferative diabetic retinopathy.

## Data Availability

Data are available on reasonable request by the contributing author.

## Supplementary Materials

The following are available online at https://www.mdpi.com/article/10.3390/ijerph18115846/s1.
